# Preventing sight-threatening ROP: the role of nurses in reducing the risk

**Published:** 2017

**Authors:** Ana Quiroga, Sarah Moxon

**Affiliations:** Neonatology Technical Advisor: National Ministry of Health and Director of Postgraduate Neonatal Nursing: Austral University, Buenos Aires, Argentina.; Research Fellow: Maternal, Adolescent, Reproductive and Child Health (MARCH) Centre, London School of Hygiene & Tropical Medicine, London, UK.

**Figure:**
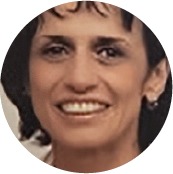
Ana Quiroga

**Figure:**
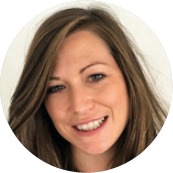
Sarah Moxon

**Neonatal nurses have frequent contact with preterm babies and their parents. By providing high quality care, nurses play an essential role in preventing retinopathy of prematurity.**

**Figure 1:**
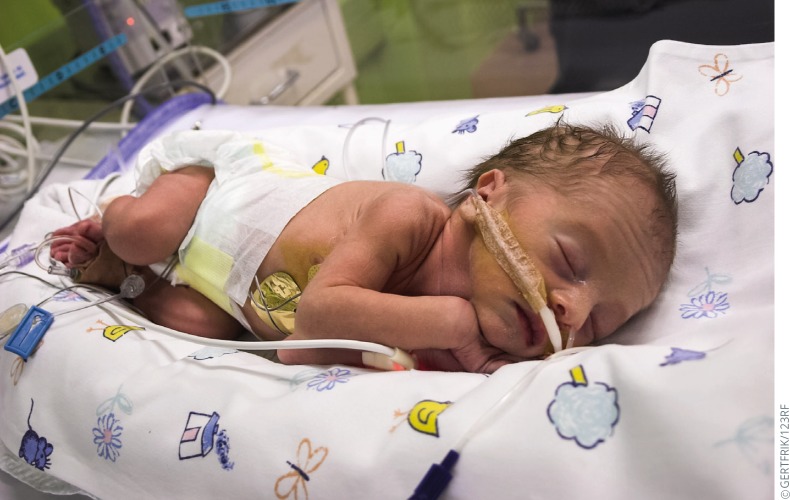
Positioning babies so they are comfortable and supported reduces stress and promotes normal neuromuscular development.

Skilled neonatal nurses play a central role as part of the multi-disciplinary neonatal team caring for preterm newborns. However, neonatal nursing is not a recognised profession in many countries, and nurses face significant challenges in providing high quality neonatal care.

Nurses can help to prevent ROP by focusing on reducing risk factors and through the day-to-day care they deliver. These are highlighted below using the POINTS of Care system (Figure 2).

**Figure 2:**
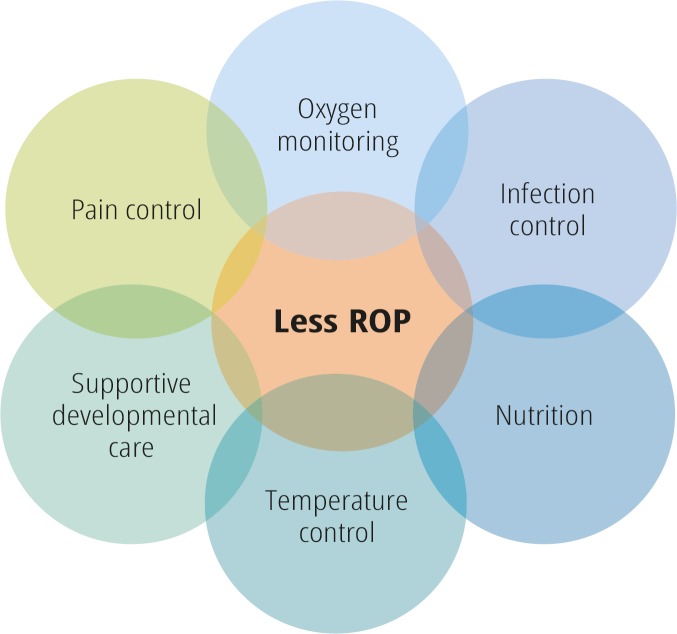
The POINTS of Care to reduce ROP

## Pain control

Procedures such as taking blood, setting up drips, or inserting a nasogastric tube are painful and can destabilise preterm babies. Painful procedures should be kept to a minimum, and pain can be reduced by giving the baby oral sucrose solution or a dummy (pacifier) to suck on before the procedure. For very painful procedures, systemic analgesics can be used.

## Oxygen monitoring

All nurses working in the neonatal unit are responsible for monitoring oxygen saturation using pulse oximeters, which is the standard of care for every newborn receiving supplemental oxygen (pp. 50–52). Nurses are responsible for ensuring that the concentration of oxygen is optimum by setting the alarms on oxygen monitors and responding quickly when they sound. Alarms must be set at 88% and 95% so that they sound if a baby's oxygen saturation falls below 89% or rises above 94%. Maintaining oxygen saturation within the targets recommended requires 24-hour care and a high level of awareness of the dangers of oxygen saturations that are too high or too low. Oxygen, compressed air, blenders, flowmeters, oxygen humidifiers, pulse oximeters, and monitors are essential items.

## Infection control

Preterm babies are much more susceptible to infection than adults and are less able to combat it. Early-onset infection (within 48 hours of birth) is usually acquired during delivery. Late-onset infection is more common and is acquired through cross-infection within the neonatal unit. The key to preventing late-onset infection is hand washing on entering the unit and before and after caring for every baby. This also applies to visitors and ophthalmologists. Other measures to prevent infection include careful skin preparation before taking blood or putting up a drip, ensuring that toys or other objects are not left in the cot, and avoiding the use of broad-spectrum antibiotics. Infection can also be reduced by keeping the neonatal unit clean and not sharing equipment, such as stethoscopes, between babies. Babies that are cared for by their mother and fed her breast milk have their gut colonised by helpful rather than harmful organisms.

**Figure 3:**
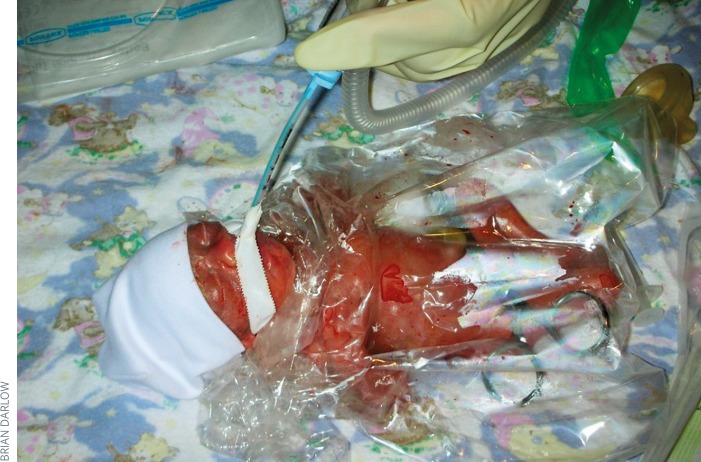
Placing a preterm baby in a plastic bag after birth is effective at maintaining normal body temperature

## Nutrition

Good nutrition is essential for the normal growth and development of preterm babies and helps to reduce the risk of infection and ROP. Preterm babies, like all other babies, need calories from fats and carbohydrates, protein, minerals and vitamins. The best food for preterm babies is their mother's own breast milk. If they are too immature to breast feed, breast milk can be given in very small amounts, within days of birth, using a small spoon, cup, or bottle. Mothers can express and store their milk in a refrigerator in the unit. Breast milk can be fortified with additional nutrients, or babies can be fed standard infant formula feeds. Intravenous nutrition is required for babies who are too immature or sick for oral feeding.

## Temperature

Preterm babies are not able to shiver if they become cold. They compensate by consuming more oxygen, which increases their oxygen requirements. Nurses can control the surrounding environment by avoiding drafts, using incubators, or by using hats and warmed cots. Plastic bags can also be used (Figure 3). Kangaroo care (continuous and prolonged skin-to-skin contact with the mother or father – see p. 48), is a nurse-led intervention which helps preterm babies to maintain their temperature within the normal range.

## Supportive developmental care

When preterm babies become stressed, their heart rate, respiratory rate and blood pressure all rise; this can lead to fluctuating oxygen saturations. Nursing care can reduce stress by reducing noise and bright lights and by positioning babies so they are comfortable and their limbs are supported (Figure 1). Nurses can reduce the number of times they disturb babies by grouping procedures together and allowing longer periods when babies are pain free, comfortable and able to sleep. Kangaroo care also helps to keep babies stable and warm, increases maternal breast milk production, encourages breast feeding, and promotes bonding between parents and their child.

### Avoiding blood transfusions and anaemia

Blood transfusion is a risk factor for ROP and unnecessary blood transfusions should be avoided. Anaemia in premature newborns is often exacerbated by taking too much blood for laboratory tests, too often. The smallest babies suffer the greatest proportional blood loss. Nurses are responsible for monitoring and limiting blood taking so that it is for critical tests only. When around 10% of total blood volume is used for blood tests, senior staff should be alerted.

### Before and during screening

Neonatal nurses are responsible for preparing preterm babies for screening, preparing the equipment needed, and caring for the babies during screening. Dilating eye drops should be administered one hour before screening is due to ensure the pupils are well dilated. During screening, the infant should be wrapped securely and given sucrose solution or a pacifier to reduce pain. Nurses are experienced at positioning babies and can minimise head movement so that screening can be done as quickly as possible, particularly if the baby is unstable or sick. Nurses should also monitor the vital signs (heart rate, oxygen saturation, etc.) throughout the procedure and ensure that the baby is stable afterwards.

## Challenges

Neonatal units in many low- and middle-income countries often have too few trained nurses. Many nurses have not had specialist training. Even in settings where nurses are trained, high staff turnover and rotation is common, leading to critical skills gaps and a lack of mentoring. Nurses cannot provide quality care if they do not have the right equipment and if there are no written policies and guidelines on safe oxygen use, for example. These factors disempower nurses and make it difficult for them to play a critical advocacy and leadership role in the planning, management, and day-today delivery of high quality neonatal care.

## Summary

Nurses play a critically important role in preventing ROP. In countries where neonatal care is relatively new, nurses may not know about ROP nor appreciate how much they can to do prevent blindness from ROP in the babies they are caring for. As eye care professionals, we can educate them about ROP by adapting our teaching approach to match their levels of knowledge and experience.
